# Dispersion of Hydrophilic Nanoparticles in Natural Rubber with Phospholipids

**DOI:** 10.3390/polym16202901

**Published:** 2024-10-15

**Authors:** Jiramate Kitjanon, Nililla Nisoh, Saree Phongphanphanee, Nattaporn Chattham, Mikko Karttunen, Jirasak Wong-ekkabut

**Affiliations:** 1 Department of Physics, Faculty of Science, Kasetsart University, Bangkok 10900, Thailand; k.jiramatez@gmail.com (J.K.); nililla.ni@ku.th (N.N.); nattaporn.c@ku.th (N.C.); 2 Computational Biomodelling Laboratory for Agricultural Science and Technology (CBLAST), Faculty of Science, Kasetsart University, Bangkok 10900, Thailand; saree.p@ku.th; 3 Thailand Center of Excellence in Physics (ThEP Center), Commission on Higher Education, Bangkok 10400, Thailand; 4 Department of Material Science, Faculty of Science, Kasetsart University, Bangkok 10900, Thailand; 5 Specialized Center of Rubber and Polymer Materials in Agriculture and Industry (RPM), Faculty of Science, Kasetsart University, Bangkok 10900, Thailand; 6 Department of Chemistry, The University of Western Ontario, 1151 Richmond Street, London, ON N6A 3K7, Canada; mkarttu@uwo.ca; 7 Department of Physics and Astronomy, The University of Western Ontario, 1151 Richmond Street, London, ON N6A 3K7, Canada

**Keywords:** coarse-grained molecular dynamics simulation, natural rubber, *cis*-1,4-polyisoprene, phospholipid, carbon nanoparticle

## Abstract

Coarse-grained molecular dynamics (CGMD) simulations were employed to investigate the effects of phospholipids on the aggregation of hydrophilic, modified carbon-nanoparticle fillers in *cis*-polyisoprene (*cis*-PI) composites. The MARTINI force field was applied to model dipalmitoylphosphatidylcholine (DPPC) lipids and hydrophilic modified fullerenes (HMFs). The simulations of DPPC in *cis*-PI composites show that the DPPC lipids self-assemble to form a reverse micelle in a rubber matrix. Moreover, HMF molecules readily aggregate into a cluster, in agreement with the previous studies. Interestingly, the mixture of the DPPC and HMF in the rubber matrix shows a cluster of HMF is encapsulated inside the DPPC reverse micelle. The HMF encapsulated micelles disperse well in the rubber matrix, and their sizes are dependent on the lipid concentration. Mechanical and thermal properties of the composites were analyzed by calculating the diffusion coefficients (*D*), bulk modulus (*κ*), and glass transition temperatures (*T_g_*). The results suggest that DPPC acts as a plasticizer and enhances the flexibility of the HMF-DPPC rubber composites. These findings provide valuable insights into the design and process of high-performance rubber composites, offering improved mechanical and thermal properties for various applications.

## 1. Introduction

Natural rubber (NR) is mostly composed of high molecular weight *cis*-1,4-polyisoprene (*cis*-PI). Its exceptional attributes in fatigue resistance, resilience, and strength have led to widespread applications, including tire manufacturing [1], high-performance plastics [2], and electrical wiring [3]. Nonetheless, NR has certain limitations in terms of low mechanical properties such as hardness, tensile strength, elastic modulus, and tear strength [4,5,6]. Consequently, there has been a substantial focus on the addition of fillers in NR to improve mechanical properties, and the other properties such as electrical and thermal properties, self-healing, and glass transition.

NR consists of *cis*-1,4-polyisoprene and non-rubber components (NRC), which include proteins, phospholipids, metal ions, carbohydrates, etc. [7,8]. NRCs are important to the mechanical properties because they induce strain-induced crystallization behavior; [9,10] the absence of NRCs leads to a significant reduction in strength and tensile properties [9,10], as well as storage modulus [11]. Interestingly, the addition of phospholipids can extend the storage time of natural rubber [12]. On the other hand, Bera et al. have demonstrated that deficiency of phospholipids in vulcanized natural rubber increased the aggregation of silica nanoparticles, leading to nonuniform dispersion [13].

Silica-filled rubber offers the benefit of reducing the rolling resistance of tire treads resulting in fuel saving tires [14,15]. In addition, silica provides a distinct combination of tear strength, aging resistance, and adhesion properties [5]. However, the hydroxyl groups in silica are hydrophilic, which results in less dispersion in hydrophobic rubbers [16]. The consequence of the strong filler–filler interactions in silica-filled rubber compounds show poor curing, low mechanical properties, and reduced thermal properties of the composites [6,17,18,19,20]. Therefore, processing techniques before vulcanization to improve the dispersion of the fillers are crucial for developing reinforcement materials. Highly filled composites exhibit high viscosity and stiffness, resulting higher mixing temperature, complicated processing, and increased production costs [21,22,23,24]. To address the issue of processing, plasticizers such as dioctylphthalate (DOP), tricresyl phosphate (TCP), dioctyl adipate (DOA), and petroleum-based plasticizer oils (including aromatic, naphthenic, and paraffinic types) [25,26], have been utilized to decrease the viscosity of dense compounds and reduce processing energy [25,26,27].

In depth understanding of silica dispersions and the effects of plasticizers in NR requires the study of aggregate structures at the molecular level. Atomistic molecular dynamics (MD) simulations are an effective method to provide the physical behavior and the interactions at the atomic scale, such as dispersion and aggregation of fillers [28], as well as alignment of rubber chains and fillers [29]. Although atomistic MD is commonly used for investigating reinforced polymer nanocomposites [28,30], it is computationally expensive. Alternatively, coarse-grained (CG) models offer a more efficient approach, providing simplified representations of polymer molecules over greater time and length scales.

The earliest polymer melt CG simulations (using Lennard–Jones-level descriptions) of polymer melts focused primarily on melts well above the glass transition temperature [31]. More CG simulations began to emerge upon the introduction of softer pairwise interactions obtained from atomistic models through pair distribution averaging [32]. Recently, CG models have been widely applied to study polymers and polymer composites [33,34,35,36,37,38,39,40]. The advantages in time and length of CG models allow studies of aggregation and dispersion of fillers [41]. Despite several existing CG models, the MARTINI force field has emerged as the most dominant, widely applied model in simulations across various molecules, including amino acids, water, phospholipid membranes, fullerenes, polymers, and RNA [42,43,44,45,46,47,48,49,50,51,52]. In our previous study, we developed and deployed a CG model of *cis*-1,4-polyisoprene (*cis*-PI) based on the MARTINI force field version 2.1 [53,54]. The model shows qualitative and quantitative agreement with experiments and atomistic simulations in which the glass transition temperature (*T_g_*) of the *cis*-PI in melts showed only a 0.5% difference with respect to the experimental result [53,55].

Herein, we performed CGMD simulations to investigate the dispersion of hydrophilic filler and lipids in the *cis*-PI matrix. Hydrophilic modified fullerenes (HMFs) were used to represent a model for a hydrophilic filler. We investigated the effects of lipid concentration on the structure, average cluster size, and radial distribution functions in the HMF-DPPC-*cis*-PI matrix. The macroscopic and thermal properties of the HMF-DPPC-*cis*-PI composite, specifically the diffusion coefficients, bulk modulus, and the glass transition temperature, were analyzed. The results show that the DPPC lipids have a significant role in the rubber-filler matrix.

## 2. Methodology

### 2.1. Molecular Dynamics Simulations

**Figure 1:**
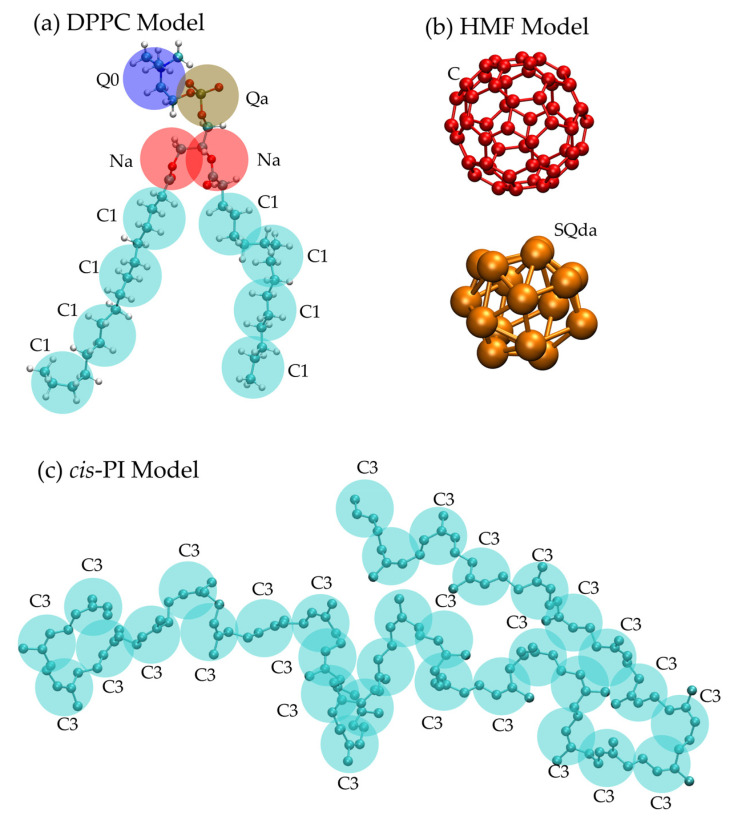
Mapping the atomistic model onto the CG model for *cis*-PI, DPPC, and HMF molecules: (**a**) DPPC model with CG bead type names Q0 (charged), Qa (charged), Na (nonpolar), and C1 (apolar) connected beads; (**b**) pristine fullerene and HMF with CG bead type names SQda (charged) interconnected beads; (**c**) rubber chain with atom type name C3 (apolar) connected to form a chain. Note that: the bead types Q0, Qa, Na, and C1 are adopted from the standard MARTINI force field [42].

MD simulations were performed using the CG MARTINI force field version 2.1 [42]. The rubber composites consisted of *cis*-polyisoprene (*cis*-PI) chains [54], dipalmitoylphosphatidylcholine (DPPC) lipids [42], and hydrophilic modified fullerenes (HMF). The structure and topology of HMF were adopted from previous studies [56,57] and the atom type SQda was applied. The polymer matrix comprised 300 CG *cis*-polyisoprene chains, each chain consisting of 32 monomers. Mapping of the atomistic model onto the CG model and CG bead types are shown in Figure 1. The numbers of DPPC and HMF introduced into the *cis*-PI composites are given in Table 1. A link to the files containing the simulation parameters and force fields is provided in the Data Availability Statement at the end of this article.

GROMACS version 5.1.1. [58] was employed in the NPT (constant number of particles, temperature, pressure) ensemble. Constant temperature was maintained (at 300 K) using the Parrinello–Donadio–Bussi velocity rescale thermostat algorithm [59,60]. The pressure was set to 1 bar using the Parrinello–Rahman algorithm [61], with a time constant of 1 ps and compressibility of 4.5 × 10^−5^ bar^−1^. Long-range electrostatic interactions were computed using the reaction field method [62,63], and the Lennard–Jones interactions were truncated at a cutoff distance of 1.1 nm. These methods have been previously validated and applied [29,53,54,64,65,66,67].

In the MD simulations, the time step was set to 20 ps and all systems were simulated for a total of 20 µs. Equilibration was monitored by following the time evolutions of the end-to-end distance and the radius of gyration (Figure S1). Data analysis was performed using the last 5 µs of each trajectory. Molecular visualizations were generated using the Visual Molecular Dynamics (VMD) software version 1.9.4 [68]

### 2.2. Calculation of Macroscopic Properties

#### 2.2.1. Bulk Modulus

The bulk modulus (*κ*) was calculated to study the DPPC concentration-dependent macroscopic properties of HMF-DPPC-*cis*-PI composites. Fluctuations of the simulation box volume (*V*) were used to calculate the bulk modulus [69] as

(1) $$\kappa =\frac{k_{B}\langle V\rangle }{\langle {\left(V-\langle V\rangle \right)}^{2}\rangle },$$

where $k_{B}$ is the Boltzmann constant and *T* is the temperature. The angular bracket refers to averaging over simulation time.

#### 2.2.2. Diffusion Coefficients

To quantify the effect of DPPC concentration on the dynamical properties of the HMF-DPPC-*cis*-PI composites, we calculated the diffusion coefficients in all directions of the *cis*-PI chains and HMF molecules in the composites using the mean squared displacement (*MSD*),

(2) $$MSD=\langle r^{2}\left(t\right)\rangle \;\sim \;6Dt,$$

where D is the diffusion coefficient, t is the time, and *r(t)* is the displacement vector of a particle. We calculated the *MSD* every 50 ns for the last 5 µs. The *MSD* between 10–40 ns in each window was fitted to calculate the diffusion coefficient.

### 2.3. Calculation of Glass Transition Temperature

To elaborate the changes in the thermal and plasticizing properties of HMF-DPPC-*cis*-PI composites, the glass transition temperatures (*T_g_*) were determined by conducting extended simulations from the final equilibrated state. In these simulations, all systems underwent a cooling process with a step size of 10 K and a time step of 100 ns, starting from 300 K and ending at 100 K, employing a cooling rate of 0.1 Kns^−1^.

For subsequent data analysis, each temperature point was subjected to an additional simulation of 500 ns in the NPT ensemble. Consequently, the total simulation time for each system amounted to 12.6 µs. *T_g_* values were ascertained by examining the change in density as a function of temperature, a method previously outlined in references [70,71,72]. Figure S4 illustrates the density versus temperature curves for both *cis*-PI in melts and composites.

## 3. Results and Discussion

### 3.1. Effect of DPPC Lipid Concentration on the Dispersion and Aggregation of HMF in cis-PI Composites

**Figure 2:**
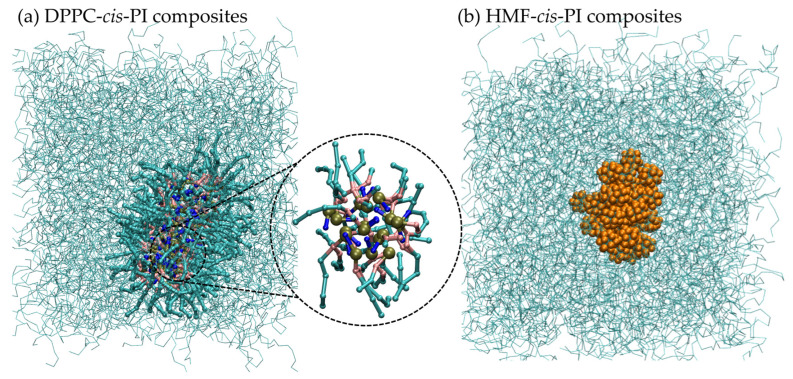
Snapshots of (**a**) DPPC-*cis*-PI composites and (**b**) HMF-*cis*-PI composites. Cyan chain: *cis*-PI chain, cyan cylinders: lipid tail, dark yellow: phosphate group, pink: glycerol backbone, blue: choline group, and orange: HMF.

Figure 2 shows the aggregation of DPPC and HMF molecules in the *cis*-PI composites (systems No. 3 and 6 in Table 1). All 80 lipid molecules form a cluster with a structure of reverse micelle (inset Figure 2a). In addition, reverse micelles were found in all concentrations of DPPC lipids in the *cis*-PI composites (Figure S2). The formation of phospholipid-based reverse micelles occurs in the hydrophobic environment because of the strong attraction of the hydrophilic head groups. [73,74,75] Similarly, the HMF particles rapidly aggregate in *cis*-PI composites (Figure 2b) and form a cluster over the simulation time. The aggregation of hydrophilic filler in the hydrophobic polymer is in agreement with previous studies. [76,77] Interestingly, a previous study by Bera et al. shows the absence of lipids in NR causing less hydrophilic particle dispersion [13].

**Figure 3:**
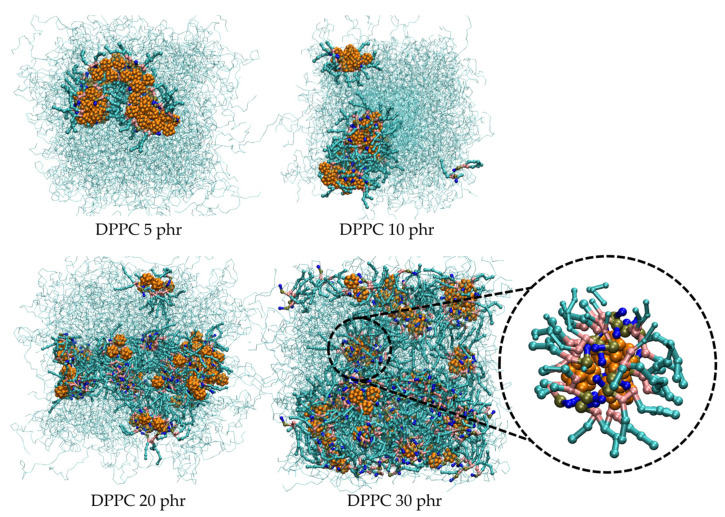
Snapshots of HMF-DPPC-*cis*-PI composites at DPPC concentrations of 5, 10, 20, and 30 phr. Cyan chain: *cis*-PI chain, cyan cylinders: lipid tail, dark yellow: phosphate group, pink: glycerol backbone, blue: choline group, and orange: HMF.

**Figure 4:**
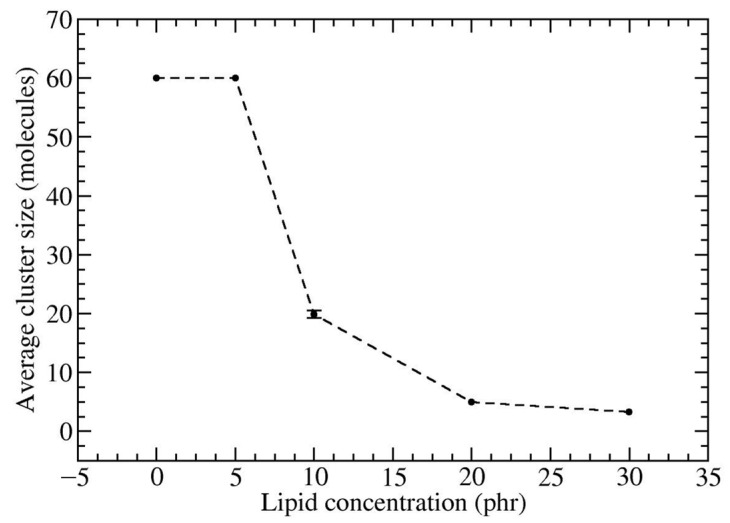
The average cluster size of HMF within DPPC-*cis*-PI composites as a function of lipid concentration.

Next, we elaborate the role of DPPC on the dispersion of HMF in a *cis*-PI matrix by varying the concentrations of DPPC. Initially, the system started from randomly distributed *cis*-PI, DPPC, and HMF within the simulation box. As the simulation time increases, the HMF molecules begin to form small clusters. Subsequently, several small clusters aggregate to form larger clusters. Concurrently, HMF clusters were adsorbed on the surface by the DPPC lipids with their hydrophilic head groups. After 100 ns, DPPC head group formed reverse micelles, completely enclosing and confining the HMF cluster within (Figure S3). Figure 3 shows the last frames from simulations of HMF-DPPC-*cis*-PI composites at various DPPC concentrations, highlighting that the reverse micelles effectively separate and encapsulate HMF clusters. The sizes of the HMF clusters decrease by 66% when the DPPC concentration is at 10 phr (as seen in Figure 4), compared to the system without DPPC. The HMF cluster size decreased by 91% and 94% with increasing DPPC concentration of 20 phr and 30 phr, respectively. Note that at low lipid concentration of 5 phr, the amount of DPPC is insufficient to cover the entire HMF cluster. This behavior suggests that the reverse micelle enhances the dispersion of HMF in rubber. The enhanced dispersion of HMF by DPPC lipids reveals the unique and noteworthy aspects of the improvement in the mechanical properties of rubber composites, such as tensile strength [78,79], tear strength [79], and rubber chain mobility [80].

**Figure 5:**
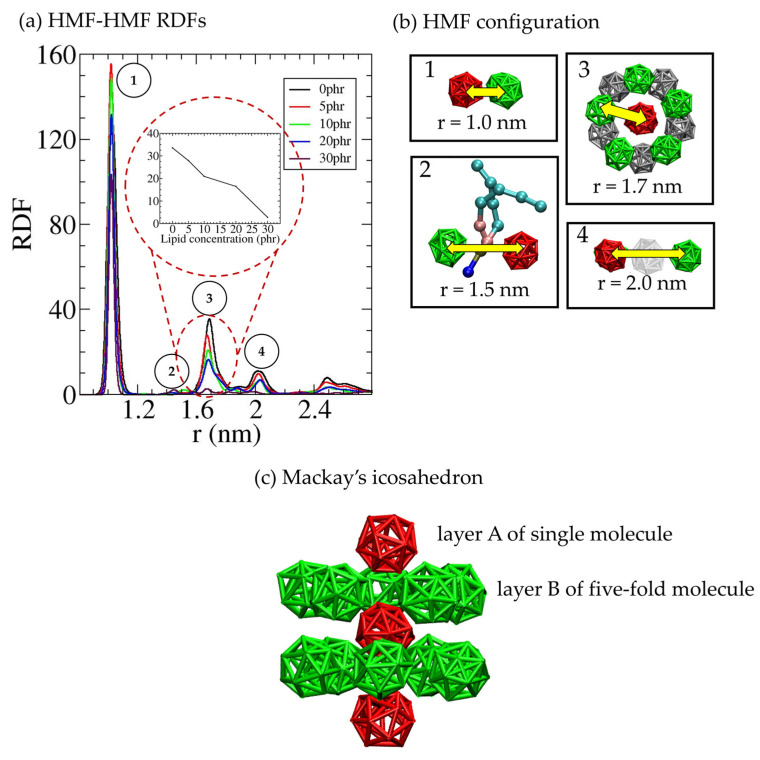
(**a**) HMF-HMF radial distribution function (RDF) within the HMF-DPPC-*cis*-PI composites. (**b**) Snapshots of representative filler configurations corresponding to the specific numbers in figure (**a**). (**c**) Side view of Mackay’s icosahedron structure.

To gain a deeper insight into the HMF cluster conformation, we calculated the center-of-mass radial distribution function (RDF) of HMF-HMF pairs while varying the DPPC concentration (Figure 5). Figure 5a shows three sharp peaks in the RDFs located at 1.0 nm, 1.5 nm, 1.7 nm, and 2.0 nm. The positions of peaks are in agreement with the interactions of fullerenes in lipid membranes [49,50,81]. Among these peaks, the highest one occurs at 1.0 nm, indicating strong interactions between the HMF particles, representing the formation of dimerized structures. This is illustrated in the HMF configuration shown in Figure 5b. A small peak at 1.5 nm at DPPC concentration of 30 phr represents the configuration of a head group of a DPPC lipid inserting between HMF particles. This configuration has been previously observed in systems of hydrophobic fullerenes in lipid bilayer and *cis*-PI composites, where the monomers were separated by either a lipid tail or *cis*-PI chain [49,53]. The third peak at 1.7 nm corresponds to the distance of the neighboring HMF in the second shell, indicating the aggregation of HMF to large clusters with Mackay’s icosahedral structure [82,83], as illustrated in Figure 5(b2). Icosahedral structures have been also observed in several studies of fullerenes [49,84,85]. This structure consists of a stack of AB layers, where layer A consists of a single molecule, and layer B has a fivefold symmetry with molecules in the same plane as shown in Figure 5c. We observed that the height of the third peak decreases when the concentration of DPPC increases, inset in Figure 5a. This result indicates that the number of close-packed large HMF clusters decreases and the dispersion of HMF becomes enhanced by the DPPC lipids. The third peak at 2.0 nm corresponds to HMF forming a third shell, where a HMF is positioned between a pair of HMF particles.

The RDF analysis revealed structural arrangements and interactions within the HMF-DPPC-*cis*-PI matrix, highlighting the influence of filler–filler interactions. These interactions can significantly reduce the mobility of filler in the composite [86], which is further examined through the diffusion coefficient to assess molecular mobility in the system.

### 3.2. Effect of DPPC Lipid Concentration on the Macroscopic Properties in HMF-DPPC-cis-PI Composites

**Figure 6:**
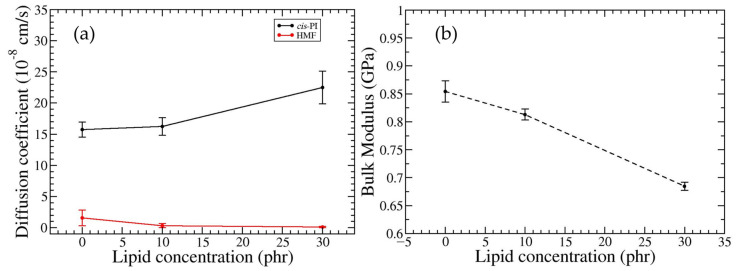
(**a**) Diffusion coefficients of *cis*-PI (black line) and HMF (red line). (**b**) Bulk modulus of HMF-DPPC-*cis*-PI composites as a function of DPPC concentration.

The strong hydrophilic interactions between the HMF molecules result in a diffusion coefficient that decreases by an order of magnitude compared to hydrophobic fullerene [53]. Additionally, with the presence of DPPC in the HMF-*cis*-PI composites, the diffusion coefficient of HMF also decreases with increasing DPPC concentration as shown in Figure 6. This result suggests that HMF is confined by the hydrophilic head groups of the reverse DPPC micelles. In contrast, the diffusion coefficient of the *cis*-PI chain increases with increasing DPPC concentration. At 10 and 30 phr of DPPC, the diffusion coefficients of *cis*-PI are increased by 4% and 42%, respectively. The increase of *cis*-PI mobility is related to the plasticizing effect, resulting in a reduction of the bulk modulus of the *cis*-PI composites (Figure 6b). Specifically, at 30 phr DPPC, the bulk modulus is decreased by 19%. These results suggest that the DPPC lipids can enhance flexibility, workability, and processability of the polymers [87], offering valuable insights for designing improved rubber composites.

### 3.3. Effect of DPPC Concentration as Plasticizer on the Thermal Properties in HMF-DPPC-cis-PI Composites

**Figure 7:**
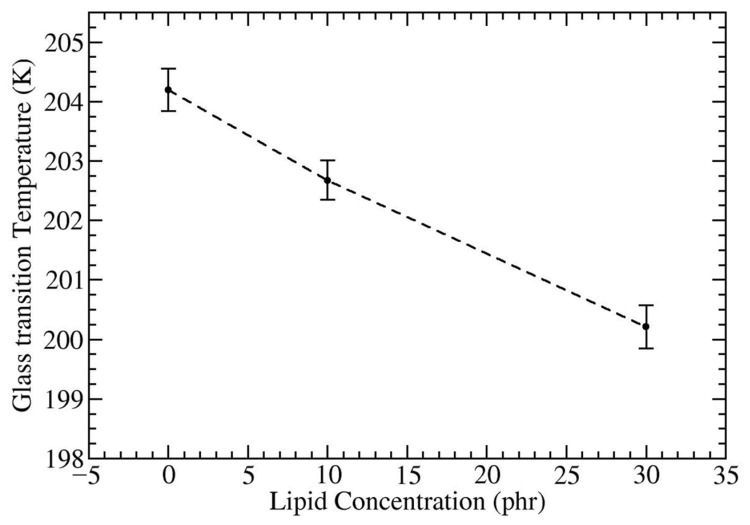
Glass transition temperature (*T_g_*) of HMF-DPPC-*cis*-PI composites as a function of DPPC concentrations.

To further explore how the DPPC concentration influences the thermal properties of these composites, it is crucial to consider the glass transition temperature. *T_g_* significantly influences the physical state and the mechanical characteristics of polymer composites and is widely recognized as a macroscopic indicator of the polymer chain’s rigidity or flexibility. The presence of the flexible polymer backbone and high polymer chain mobility leads to faster relaxation and consequent lower glass transition temperature. [88,89,90,91,92] In this study, we determined *T_g_* by analyzing the change in slope of density (*ρ*) versus temperature (*T*) [70,71,72]. Figure S4 illustrates the *ρ*-*T* curves for both *cis*-PI in melts and composites. The calculated *T_g_* of *cis*-PI in melt was found to be 201.2 ± 0.8 K, demonstrating a strong agreement with the previous results obtained by all-atom model (209 K) [72], united-atom models (223 K) [72], and experiments (200 K) [93]. With the presence of HMF, we found that the *T_g_* of HMF-*cis*-PI composite is 204.2 ± 0.4 K, which is 3.0 K higher than that of pure *cis*-PI (Figure 7). This finding aligns well with the addition of the hydrophobic fullerenes to a *cis*-PI matrix [53]. In contrast, the addition of DPPC lipids into the HMF-*cis*-PI composite at concentrations of 0–30 phr decreases *T*_g_ by 0.85% and 2% at 10 and 30 phr DPPC concentrations, respectively. The observed decrease in *T*_g_, attributed to DPPC acting as a plasticizer, supports its role in enhancing chain mobility and facilitating easier rubber processing, consistent with established plasticizer-induced modifications in rubber systems [89,94,95,96].

## 4. Conclusions

In this study, CGMD simulations using the MARTINI force field over the total time greater than 200 microseconds were employed to investigate the influence of DPPC concentrations of 0, 5, 10, 20, and 30 phr on the dispersion and aggregation of HMF fillers in *cis*-PI composites. Our simulations demonstrate that the interaction mechanisms between hydrophilic particles and lipids in rubber matrices primarily involve the formation of reverse micelles by the phospholipids in the rubber matrix. These micelles encapsulate the HMF, which would otherwise tend to aggregate due to their hydrophilic nature in the hydrophobic rubber environment. The hydrophilic head groups of the DPPC lipids attract and surround the HMF particles, leading to the formation of reverse micelles. These micelles effectively isolate and disperse the HMF particles within the rubber matrix. As the concentration of DPPC increases, the size of the HMF clusters decreases significantly by 94% at 30 phr and results in a decrease of the third HMF-HMF peak in the RDF in DPPC-*cis*-PI. The study shows that the encapsulation by the DPPC lipids reduces filler–filler interactions, thereby improving the dispersion of the hydrophilic particles and enhancing the mechanical properties of the rubber composite [19,20]. Furthermore, DPPC was found to be an effective plasticizer, increasing the diffusion coefficient by 42%, reducing the bulk modulus by 19%, and lowering *T_g_* by 2% at 30 phr DPPC concentration in HMF-DPPC-*cis*-PI. The presence of DPPC as a plasticizer is essential for rubber processing as it results in a composite material that is easier to process [25,26,27]. These insights provide a deeper understanding of the influence of DPPC, which not only improves the dispersion of hydrophilic particles in rubber matrices but also acts as a plasticizer, enhancing the processing of high-performance rubber composites for various industrial applications [19,20,25,26,27].

## Figures and Tables

**Table 1:** Simulation system details.

No.	DPPC Concentration (phr)	Molecules			Simulation Time (μs)
		*cis*-PI	DPPC	HMF	
1	0	300	0	-	20
2	5	300	40	-	20
3	10	300	80	-	20
4	20	300	160	-	20
5	30	300	240	-	20
6	0	300	0	60	20
7	5	300	40	60	20
8	10	300	80	60	20
9	20	300	160	60	20
10	30	300	240	60	20

## Data Availability

Parameters are available at DOI: https://doi.org/10.5281/zenodo.13890110 (accessed on 4 October 2024).

## Supplementary Materials

The following supporting information can be downloaded at: https://www.mdpi.com/article/10.3390/polym16202901/s1, Figure S1: Time evolution of the end-to-end distance (black line) and the radius of gyration (red) of the *cis*-PI-HMF-DPPC composites at different DPPC concentrations from 0 to 30 phr.; Figure S2: Snapshots at (a) 5 phr, (b) 20 phr and (c) 30 phr DPPC concentrations in *cis*-PI-DPPC composites.; Figure S3: (a) Visualizations of *cis*-PI-DPPC-HMF composites at 10 phr DPPC concentration: initial structure, 5.5 ns, 8 ns, 100 ns and the last frame at 20 μs. (b) The number of HMF clusters, and (c) the average cluster size of HMF at 10 phr DPPC concentration as a function of time; Figure S4: Density versus temperature of the *cis*-PI in melt and the *cis*-PI-HMF-DPPC composites.
