# Supplementary material for: Dispersion of Hydrophilic Nanoparticles in Natural Rubber with Phospholipids
Source: Polymers (Basel). 2024 Oct 15;16(20):2901. doi: 10.3390/polym16202901 (PMC11510818; doi:10.3390/polym16202901)
Supplement: Supplementary file 1 [file polymers-16-02901-s001.zip › polymers-3236058-supplementary.pdf]

Dispersion of hydrophilic nanoparticles in natural rubber with phospholipids

Jiramate Kitjanon[1,2], Nililla Nisoh[1,2,3], Saree Phongphanphanee [2,3,4,5], Nattaporn Chattham[1], Mikko Karttunen[6,7], Jirasak Wong-ekkabut[1,2,3,5*]

[1]Department of Physics, Faculty of Science, Kasetsart University, Bangkok 10900, Thailand

[2]Computational Biomodelling Laboratory for Agricultural Science and Technology (CBLAST), Faculty of Science, Kasetsart University, Bangkok 10900, Thailand

[3]Thailand Center of Excellence in Physics (ThEP Center), Ministry of Higher Education, Science, Research and Innovation, Bangkok 10400, Thailand

[4]Department of Material Science, Faculty of Science, Kasetsart University, Bangkok 10900, Thailand

[5]Specialized center of Rubber and Polymer Materials in agriculture and Industry (RPM), Faculty of Science, Kasetsart University, Bangkok, 10900, Thailand;

[6]Department of Chemistry, The University of Western Ontario, 1151 Richmond Street, London, Ontario, Canada N6A 3K7

[7]Department of Physics and Astronomy, The University of Western Ontario, 1151 Richmond Street, London, ON, Canada N6A 3K7

*E-mail addresses: jirasak.w@ku.th

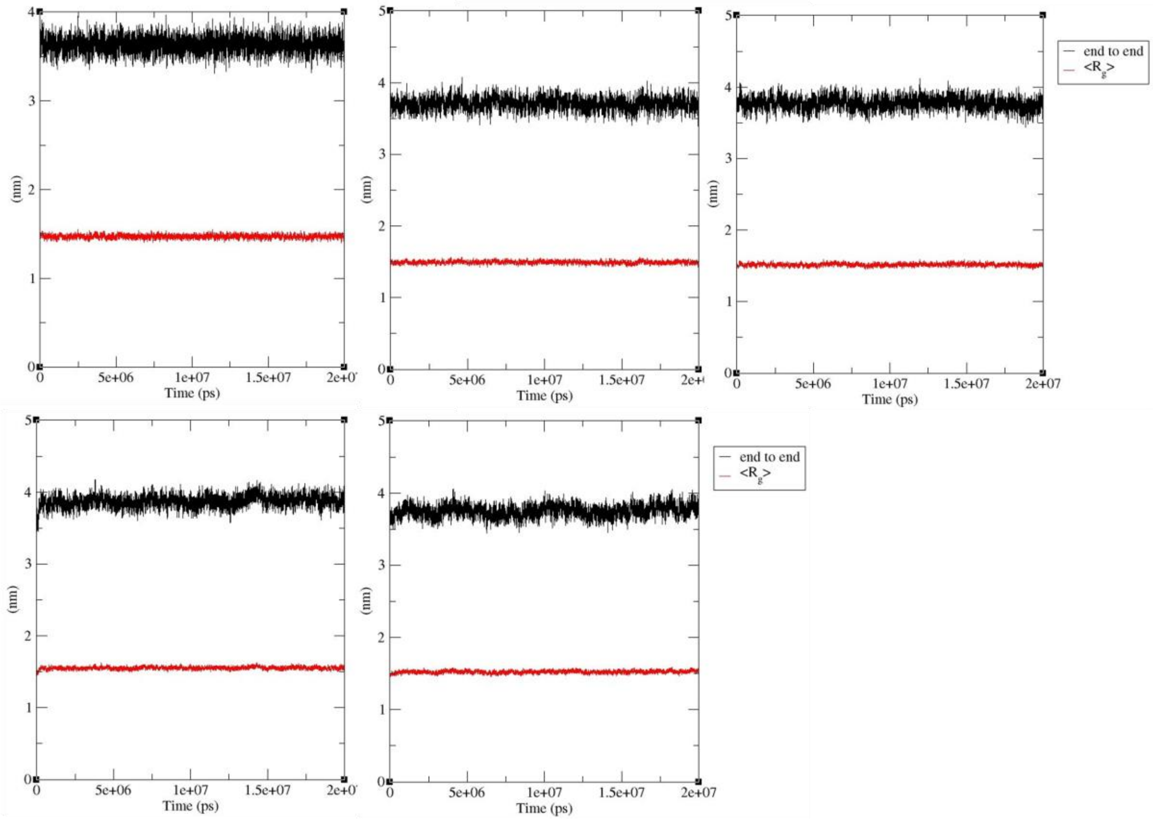

Figure S1: Time evolution of the end-to-end distance (black line) and the radius of gyration (red) of the *cis*-PI-HMF-DPPC composites at different DPPC concentrations from 0 to 30 phr.

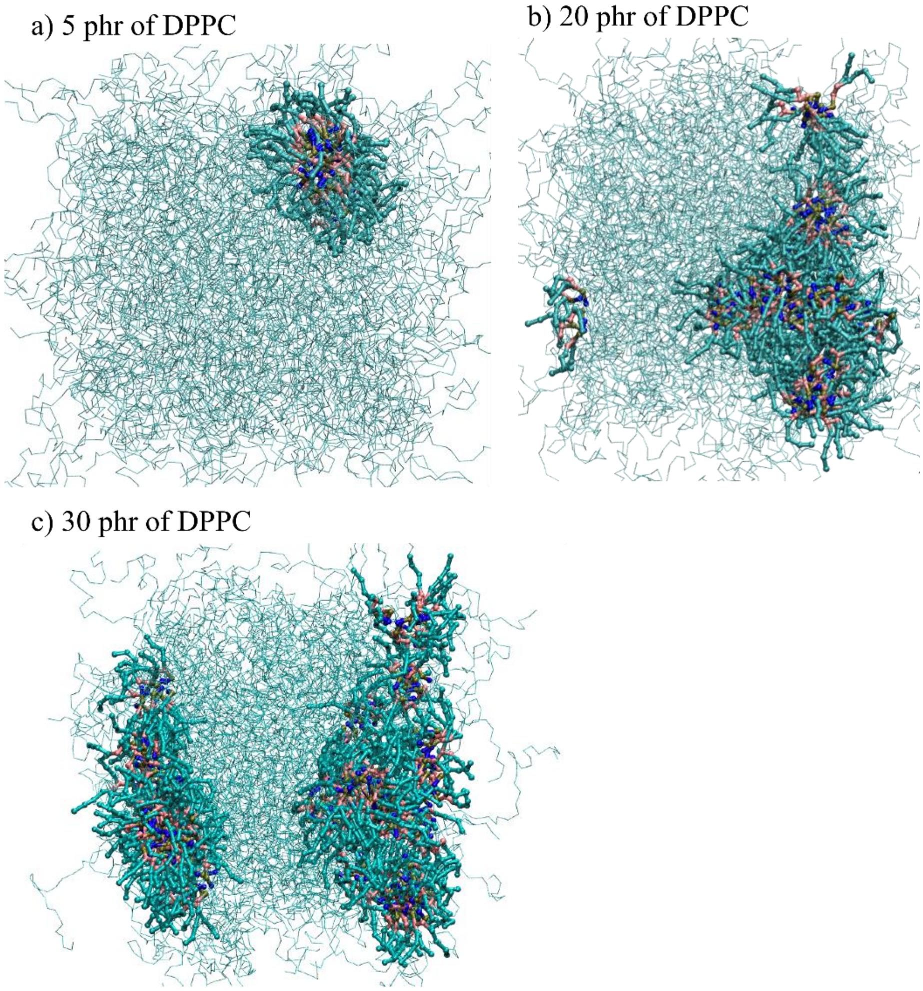

Figure S2: Snapshots at a) 5 phr, b) 20 phr and c) 30 phr DPPC concentrations in *cis*-PI-DPPC composites.

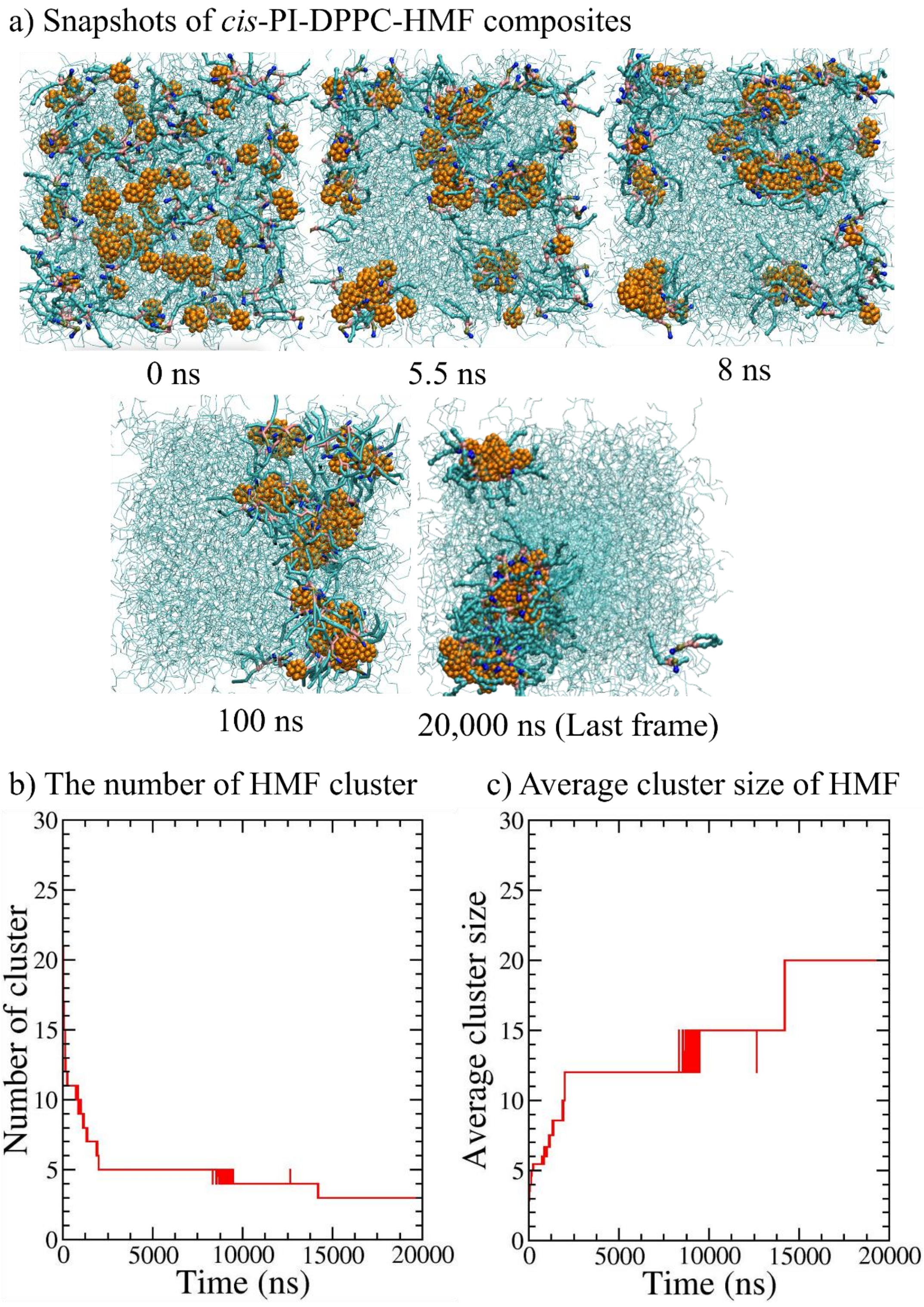

Figure S3. a) Visualizations of *cis*-PI-DPPC-HMF composites at 10 phr DPPC concentration: 0 (initial structure), 5.5, 8, 100 and 20000 (the last frame) ns. b) The number of HMF clusters, and c) the average cluster size of HMF at 10 phr DPPC concentration as a function of time.

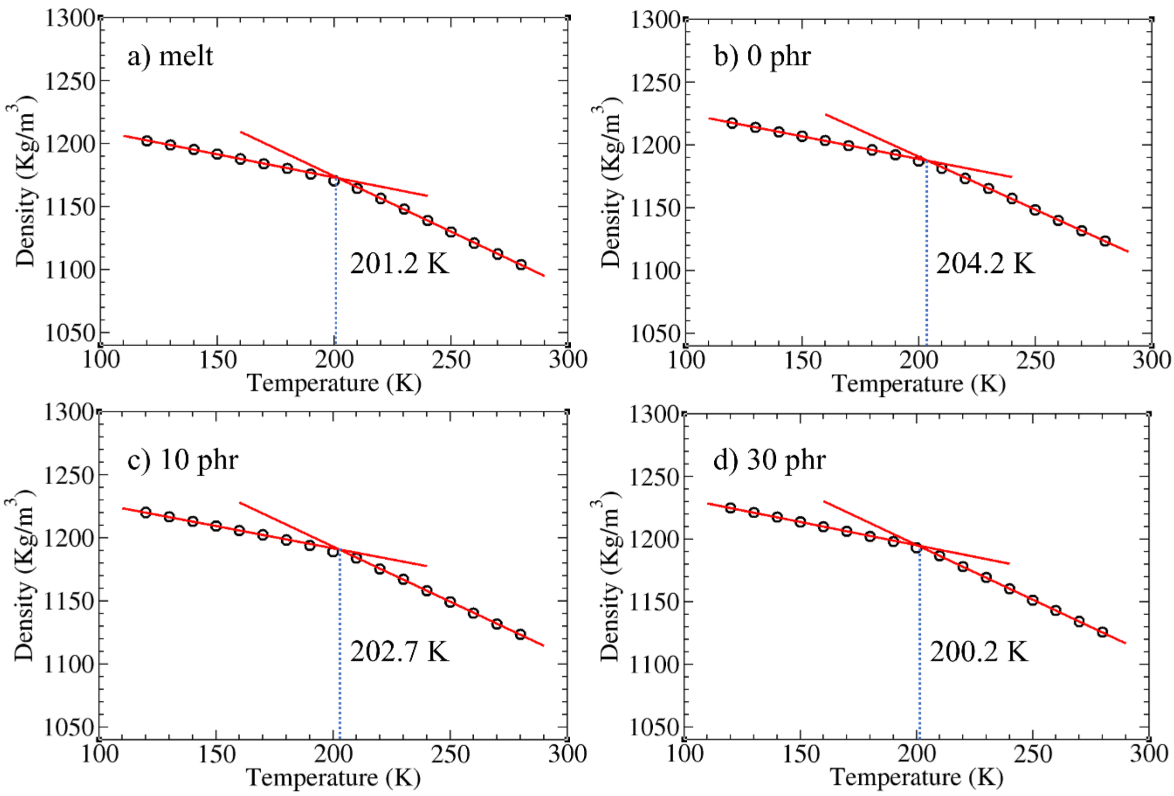

Figure S4: Density versus temperature of the *cis*-PI in melt and the *cis*-PI-HMF-DPPC composites.
